# Establishing Synoptic Cancer Pathology Reporting in Low- and Middle-Income Countries: A Nicaraguan Experience

**DOI:** 10.1200/JGO.19.00343

**Published:** 2022-02-14

**Authors:** Darling Valverde L, Richard C. Reznichek, Magdaly Torres S

**Affiliations:** ^1^ Department of Pathology, Universidad Nacional Autónoma de Nicaragua, León, León, Nicaragua; ^2^ Department of Urology, David Geffen School of Medicine at UCLA, Los Angeles, CA

## Abstract

Cancer pathology reports have traditionally been narrative with limited reliable information. In high-income countries, synoptic format reporting has gradually been introduced to improve patient care and cancer registry input. We posited that introduction of synoptic style reporting was possible in Nicaragua and could serve as a model for other low- and middle-income countries.

**MATERIALS AND METHODS:**

The Department of Pathology, Hospital Escuela Oscar Danilo Rosales Argüello (HEODRA), León, Nicaragua, decided to introduce and implement synoptic reporting for all cancer cases beginning in 2018. All 10,012 histopathologic case reports issued by the department from January 1, 2018, through June 30, 2020, were reviewed. After excluding benign lesions, recurrent or metastatic tumors, endometrial biopsies or curettage, and primary cytologic specimens, 724 cases met the criteria for synoptic style reporting. The narrative format, previously used for all cases, was intentionally abandoned.

**RESULTS:**

Of the 10,012 reports reviewed at HEODRA during the study period, synoptic-style reporting was used for all 724 cancer cases that met criteria for inclusion in the study. In addition, all elements were listed in the required order. Narrative format of reporting was not used for any of the reports.

**CONCLUSION:**

Our experience in Nicaragua has shown that establishing synoptic-style cancer pathology reporting is achievable in a low- or middle-income country. Just as in high-income countries, a dedicated collaborative step-by-step conversion to synoptic reporting can lead to improvement in cancer patient care and quality of data for population-based registries.

## INTRODUCTION

A cancer pathology report is the final written product of a surgical pathology laboratory with information crucial for patient care and cancer surveillance. Traditional narrative reports contain text that describes information in relevant headings: macroscopic description, microscopic description, and final diagnosis. These descriptive free-text reports, however, show significant variability in style and content, do not contain all clinically important data, and can cause erroneous decisions.^1^

**CONTEXT**

**Key Objective**
Is it possible to establish synoptic cancer pathology reporting in a low- or middle-income country?
**Knowledge Generated**
In a Nicaraguan public hospital, there were 10,012 histopathologic cases during the study period. Of these, 724 met the criteria for use of organ-specific cancer pathology reporting protocols. Upon review, it was found that every report used the newly introduced synoptic format. Benefits of conversion from limited narrative to structured synoptic cancer pathology reporting include accurate documentation with consistent format, elimination of transcription errors, and concise information available for treatment options and research.
**Relevance**
Synoptic cancer reporting is now established in Nicaragua, confirming that it is possible in a low- or middle-income country. This conversion to synoptic from narrative reporting can serve as a model to improve cancer patient care and the quality of data for population-based cancer registries in other countries.

Synoptic reporting is a clinical documentation method that has developed over recent decades. In the 1980s, the use of templates, protocols, and checklists became common in a variety of areas of medicine and were associated with improved communication and completeness of tasks. Hutter,^2^ in 1984, pointed out that the pathologist's documentation of breast biopsies and mastectomies can provide useful information for the selection of treatment and provide information to estimate prognosis and determine the need for adjunctive therapy. Markel and Hirsch^3^ in 1991 were first to report replacing the narrative format with the synoptic style format in their pathology department. Practice protocols for pathology were introduced and standardized in the next few years.^4,5^ Leslie and Rosai^6^ recommended that synoptic templates be used for standardization of pathology reports, with the goal of attaining uniform and consistent data relevant to clinical management of patients.

Concise, accurate tumor reporting is accomplished through the consistent use of element-based synoptic protocols. In 1998, the College of American Pathologists (CAP) introduced the first synoptic cancer-reporting guidelines for use by practicing pathologists. Multidisciplinary task forces began to release individual protocols for a variety of types of cancers. One for gastric carcinoma serves as an example.^7^ Two years later, CAP published information regarding the process of their development and approval.^8^ The protocols were designed for patient care in all types of practice settings and to assist registrars and governing bodies in the uniform collection of pathology data. Protocols include the TNM classification system, used primarily in solid tumors. Synoptic elements, including classified TMN staging, show the extent of disease. Cancer staging with TNM aids prognosis, improves communication between providers, and allows for better information sharing and research across populations.^9^ The International Classification of Diseases for Oncology (ICD-O), a standard coding tool for cancer registries, is incorporated. In June 2021, the release of updated CAP Cancer Protocols included 87 revised and three new protocols.^10^

Collection of data regarding individual tissue specimens is obtained using protocol templates in a question and answer format. For each template, required data items are followed by a list of possible appropriate responses. Templates may also include optional data items. Each diagnostic parameter pair (data item and response) must appear on a separate line to achieve visual separation. The number of parameters that a pathologist needs to include in a report depends on the organ and specimen type. Table 1 shows English and Spanish versions of an acceptable synoptic pathology report. Table 2 shows an example of a synoptic report that clearly delineates TNM staging. Conversely, Table 3 shows an example of a report that is unacceptable, in part due to the absence of TNM.

**TABLE 1:**
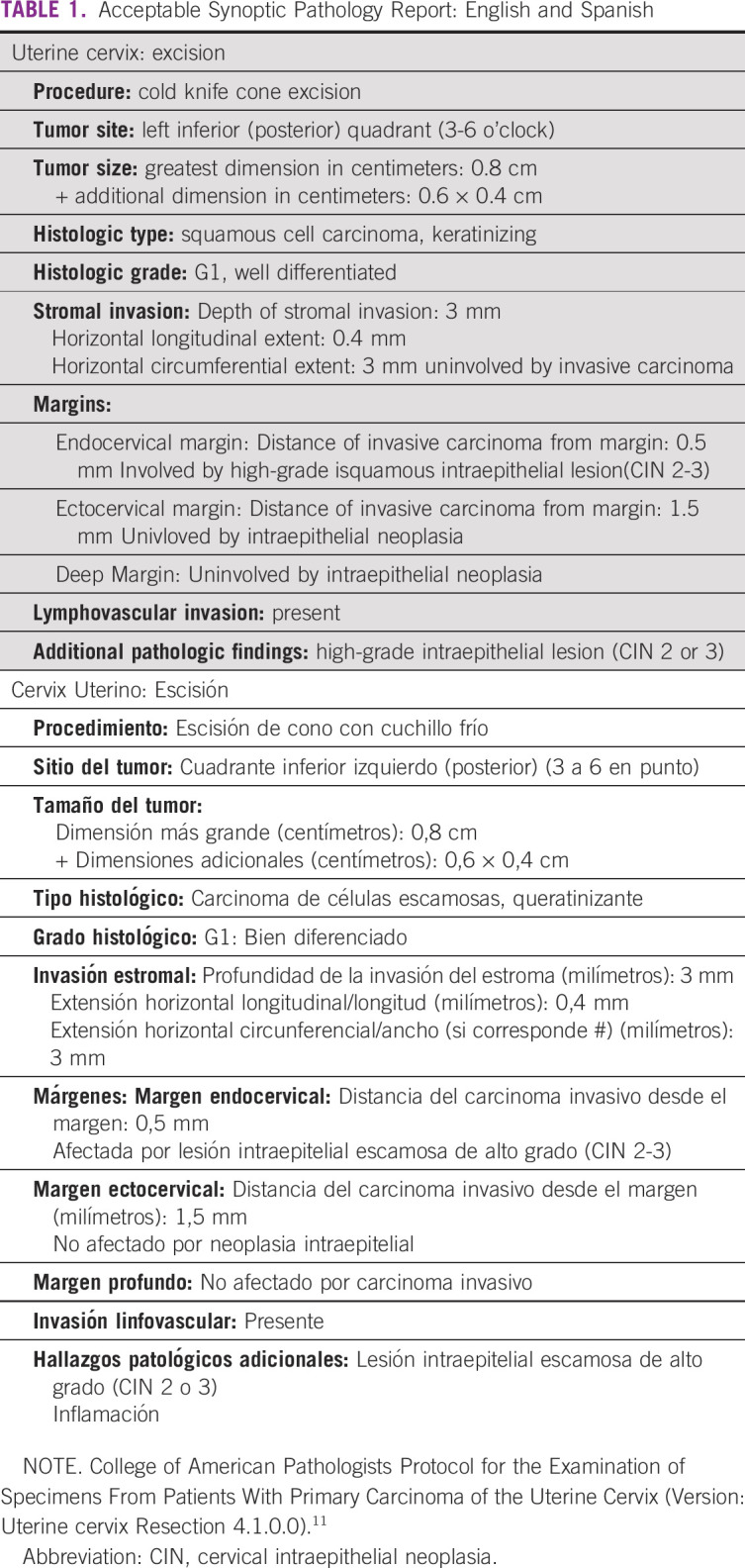
Acceptable Synoptic Pathology Report: English and Spanish

**TABLE 2:**
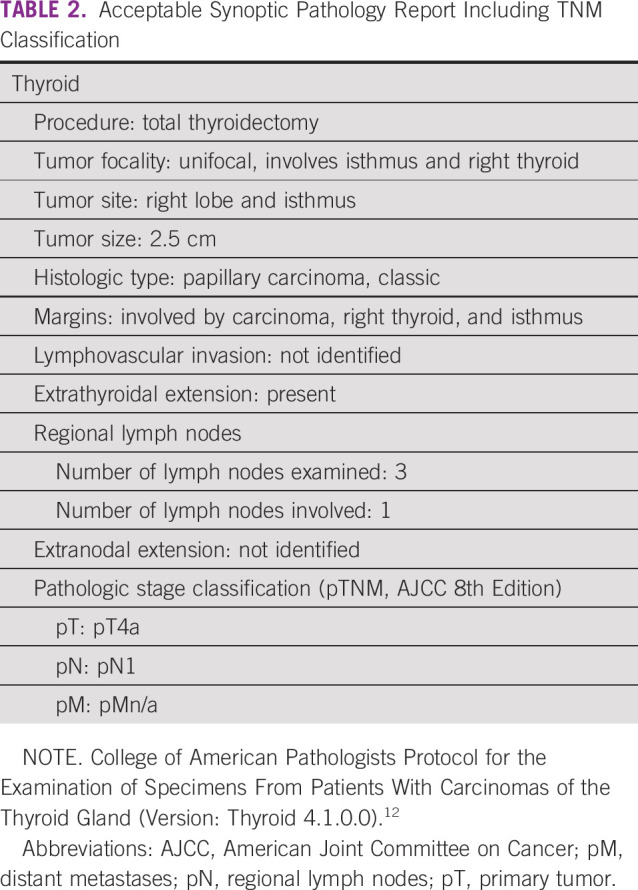
Acceptable Synoptic Pathology Report Including TNM Classification

**TABLE 3:**
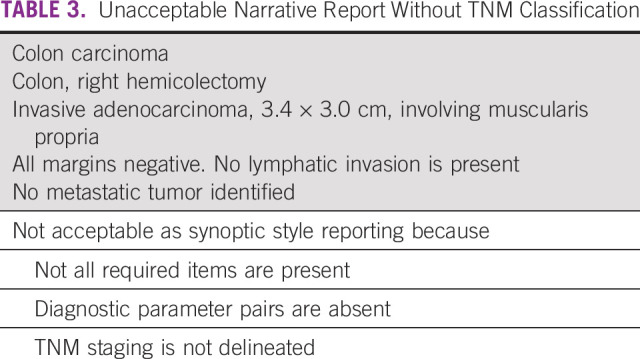
Unacceptable Narrative Report Without TNM Classification

## MATERIALS AND METHODS

All pathologists in Nicaragua had traditionally and exclusively used the narrative form of pathology reporting. The adoption of synoptic reporting in pathology practice was contemplated as part of a nation-wide system to capture and report cancer data.

Significant milestones of the development process for the entire country, beginning in 2015, are outlined in Table 4. Initially, the authors explored the concept of synoptic reporting with interested pathologists and oncologists at national meetings. Positive feedback led to discussions with the national Ministry of Health (MINSA). After receiving permission from CAP, translations into Spanish were carried out by 11 pathologists. The translated protocols were approved by a committee of experts of MINSA. In 2017, authorization to proceed with implementation was obtained from MINSA. A successful 3-month trial of reporting breast cancer cases was carried out in one hospital. Training sessions were carried out for all public sector pathologists. Starting in 2018, several hospitals in Nicaragua began using synoptic reporting for some types of cancer cases. By 2019, all pathologists had been trained in the use of protocols, and all hospitals in Nicaragua became qualified to accept them for cancer reporting. The ongoing process includes translating CAP updates, including the most recent TNM staging, and providing continuing education.

**TABLE 4:**
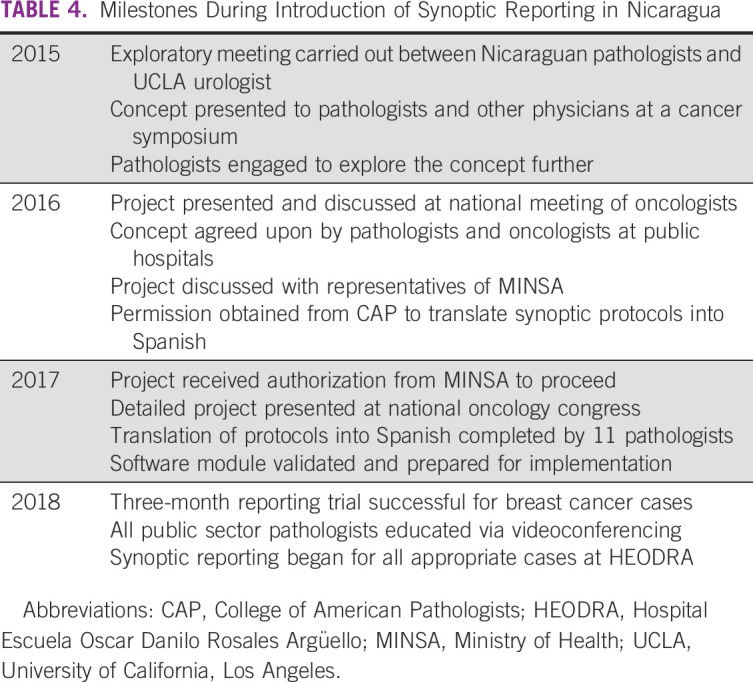
Milestones During Introduction of Synoptic Reporting in Nicaragua

The Department of Pathology, Hospital Escuela Oscar Danilo Rosales Argüello (HEODRA), León, Nicaragua, decided to establish and implement synoptic reporting for all appropriate cancer cases beginning in January 2018. The process from microscope to signed synoptic report utilized at HEODRA follow.

Registering of data begins in the operating or procedure room. The patient is identified, a unique national code is assigned, a procedure is performed, and a surgical specimen is obtained. These initial data are entered into a software program for surveillance and prevention of cancer called Sistema de Vigilancia y Prevención del Cáncer (SIVIPCAN). The second step in registration occurs when the specimen is received in the Department of Pathology where the case is assigned a hospital code. The pathologist receives the case slides along with a paper request form, examines the tissue, and handwrites a report using a synoptic protocol for appropriate cancer cases. When an element of data cannot be responded to because of the type of specimen, the reported response is not applicable or not available. A brief reason is written in parentheses (eg, not possible to specify the state of margins because of fragmented specimen). A secretary or clerk types the pathology report that the pathologist then reviews, corrects as needed, approves, and signs. Though free text is never allowed within the synoptic report, it may appear separately after the formal report. Data elements of the protocol are entered into SIVIPCAN. Copies of the report are printed for the clinical physician and the pathology archives.

All 10,012 histopathologic case reports issued by the Department of Pathology, HEODRA, from January 1, 2018, through June 30, 2020, were reviewed.

Benign cases, as well as recurrent tumors, endometrial biopsies, endometrial curettages, and primary resection cytologic specimens, were excluded. Since synoptic CAP reports are designed for primary tumors, metastatic tumor cases were excluded when surgery for the primary lesion had been performed in another hospital or when the patient had died without a histopathologic report of a primary tumor. As no cancer biomarkers were available at HEODRA, it was not possible to include that type of protocol. After exclusions, 724 cancer cases met criteria for synoptic style examination and reporting using CAP protocols.

## RESULTS

All 724 synoptic-style cancer pathology reports issued by the Department of Pathology, HEODRA, from January 1, 2018, through June 30, 2020, were reviewed. Each case report was analyzed to assess completeness of synoptic reporting. All (100%) were found to be in the synoptic format without deficiency of required data items. None of the 724 cases were in the narrative style. In addition, in every report, all elements were listed in correct order for the specific protocol template used.

Topographic distribution was based on the anatomical site of origin (Table 5). The 10 most common types of cancer seen at HEODRA, in descending order, were as follows: uterine cervix 338 (46.7%), breast 90 (12.4%), skin 82 (11.3%), colon 39 (5.4%), lymph nodes 25 (3.5%), thyroid 24 (3.3%), corpus uteri 20 (2.8%), stomach 13 (1.8%), ovary 11 (1.5%), and kidney 10 (1.4%). Patient age was distributed by age groups (Table 6). The 15- to 39-year-old age group had the largest number and percentage of synoptic case reports: 270 (35.9%). Diagnostic and demographic data for all 724 cases were submitted to SIVIPCAN, the national database.

**TABLE 5:**
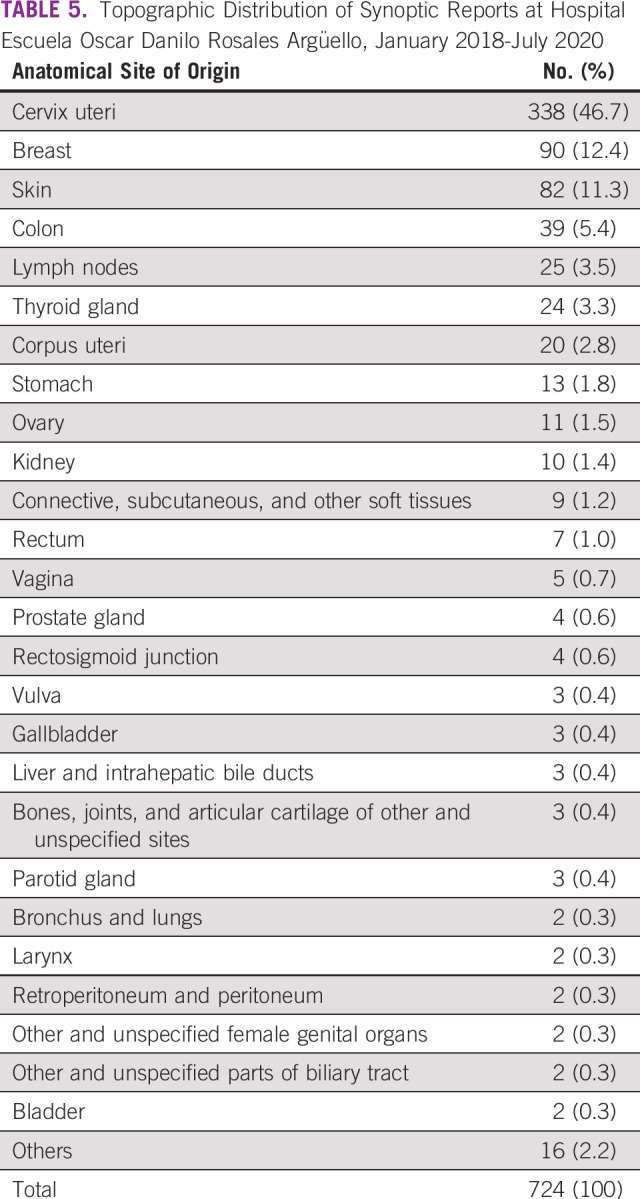
Topographic Distribution of Synoptic Reports at Hospital Escuela Oscar Danilo Rosales Argüello, January 2018-July 2020

**TABLE 6:**
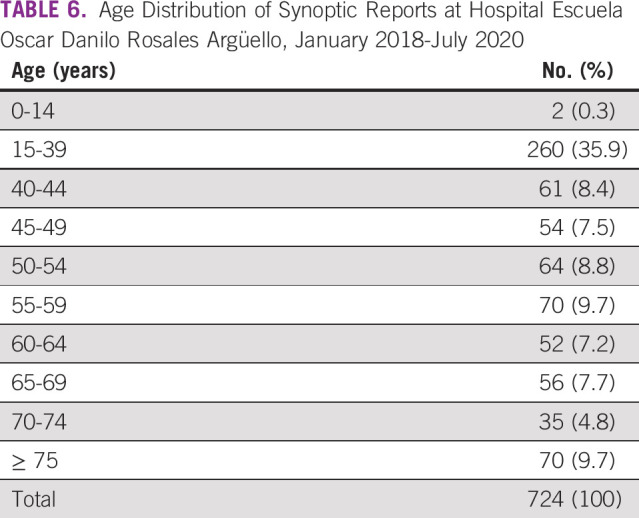
Age Distribution of Synoptic Reports at Hospital Escuela Oscar Danilo Rosales Argüello, January 2018-July 2020

## DISCUSSION

By 2030, the United Nations Sustainable Development Goal 3 seeks to reduce premature mortality from noncommunicable diseases, including cancer, by one third.^13^ More than 60% of the world's cancer cases occur in Africa, Asia, and Central and South America, and these regions account for about 70% of the cancer deaths.^14^ The president of the International Association of Cancer Registries noted that in low- and middle-income countries (LMICs), registry coverage with high-quality data remains well below 10% in Africa, Asia, and Latin America.^15^ In addition to accurate demographic information, registries need to obtain detailed case data from clinicians and pathologists. Essential variables include the following: personal identifiers and demographics, tumor biopsy or resection reports, initial therapy, and case follow-up. Registry data can then be used for selection of patient treatment options, epidemiologic studies, and public health planning. In Nicaragua, the desire for a national cancer registry to provide accurate information for clinicians and public health planning was the initial impetus to seek a more advanced level of cancer reporting.

The principal benefits of conversion from narrative to structured synoptic cancer pathology reporting include accurate documentation without variability in format, elimination of transcription errors, concise information available for treatment options and research, and robust reliable data for population-based registry and public health planning. Standardized population-based synoptic cancer pathology reports have improved support for clinical decision making, research, and clinician satisfaction.^16,17^

The value of a national approach to structured reporting is well delineated in a report to the Australian Government Department of Health by the Royal College of Pathologists of Australasia.^18^ It recognized that synoptic reporting contributes to cancer control at levels of clinical management, notification and registration, aggregated analyses, and clinical research. In addition to CAP, other national pathology groups, including the Royal College of Pathologists, have released standards and data sets.^19^ The International Collaboration on Cancer Reporting has also been developing data sets (in English, with some available in Spanish, French, and Portuguese) that provide an evidence-based approach for reporting many individual cancers.^20^

Although synoptic pathology reporting is in common use in high-income countries, the only previous study we could find in a LMIC was an investigation in Nigeria limited to comparing narrative versus synoptic reporting for prostate needle biopsies.^21^ Our experience in Nicaragua is unique since synoptic protocols were used to report all cancer cases for which there are designated CAP protocols.

Though it would have been interesting to compare completion of synoptic with previous narrative style reporting, permission could not be obtained to carry out a review of previous archived narrative reports. The two styles were never used at the same time. Implementation of synoptic reporting for all cases occurred after approval of the new platform.

Evaluation of the cases at HEODRA revealed areas for improvement that could speed the process overall, minimize errors, and maximize the use of enhanced information. These include introducing special checklists for cancers of greatest frequency, entering data directly into the computer instead of handwriting initial reports, moving to electronic reporting with drop-down menus, and promoting the use of data by clinicians and tumor boards. In addition, availability of immunohistochemistry would allow inclusion of biomarkers in reporting.

Synoptic reporting is now also being carried out by pathologists in all public hospitals in Nicaragua. For each case, demographic data, synoptic elements (including classified TNM staging), and other diagnostic data (from ICD-O catalogs) are entered into SIVIPCAN, the national database. This information can be reviewed on the network from any hospital in the country. For clinicians, the synoptic protocols used by pathologists are providing more complete data for consideration of patient treatment options. For registrars and researchers, these data provide more reliable information for epidemiologic studies and public health planning.

Resulting data, however, are not entered in a formal cancer registry that allows it to be shared and analyzed nationally. Currently, it is not possible to access precise information on the percentage of cases that receive care at individual public or private hospitals. An optimal strategy would be to have reports in a computerized mode that would allow them to be dispersed rapidly for use by consultants in real time for diagnosis, treatment planning, and surveillance. The goal is to be able to provide these data to all clinics, hospitals, and cancer care physicians, as well as the national public social security system. This capacity awaits the establishment of a national cancer registry.

In conclusion, conversion from narrative to synoptic reporting, as reported here, has inaugurated a valuable system change in the care of Nicaraguan patients with cancer. Early acceptance by local physicians and health officials led to a heartening spirit for collaboration and easy sharing of information. Even at this early stage in implementation, it provides complete, uniform, and reliable information essential for decision making, especially in regard to further diagnostic evaluation and appropriate treatment. Data from reports are submitted electronically to the national population-based data registry. Accurate reporting flows from the pathologist's desk to the cancer care clinician.

We acknowledge that the change from narrative to synoptic reporting can have limitations. LMICs that do not yet have hospitals or centers that care for patients with cancer, or are not public health-oriented, may have to await increased interest or resources.

As far as is known, the introduction of synoptic cancer pathology reporting in all public hospitals in Nicaragua is a first for a LMIC. On the basis of our successful experience, establishment of synoptic cancer reporting is recommended for all LMICs at a level that is achievable and affordable. As exemplified by our case, the lack of financial means to install an electronic synoptic software program should not delay basic conversion from narrative to synoptic style. Transition to an electronic format can occur as resources become available. With a dedicated collaborative step-by-step approach, successful establishment of synoptic reporting can significantly improve the quality of cancer patient care and the quality of data in population-based cancer registries.

## References

[1] Zarbo RJ: Interinstitutional assessment of colorectal carcinoma surgical pathology report adequacy. A College of American Pathologists Q-Probes study of practice patterns from 532 laboratories and 15,940 reports. Arch Pathol Lab Med 116:1113-1119, 1992. 1444738

[2] Hutter RV: Pathological parameters useful in predicting prognosis for patients with breast cancer. Monogr Pathol 25:175-185, 1984 6377048

[3] Markel SF, Hirsch SD: Synoptic surgical pathology reporting. Hum Pathol 22:807-810, 1991 1869264 10.1016/0046-8177(91)90209-8

[4] Ruby SG, Henson DE: Practice protocols for surgical pathology. A communication from the Cancer Committee of the College of American Pathologists. Arch Pathol Lab Med 118:120-121, 1994 8311647

[5] Scully RE, Henson DE, Nielsen ML, et al: Practice protocol for the examination of specimens removed from patients with ovarian tumors**:** A basis for checklists. Cancer 78:927-940, 1996 8756391 10.1002/(SICI)1097-0142(19960815)78:4<927::AID-CNCR33>3.0.CO;2-V

[6] Leslie KO, Rosai J: Standardization of the surgical pathology report: Formats, templates, and synoptic reports. Semin Diagn Pathol 11:253-257, 1994 7878300

[7] Compton C, Sobin LH: Protocol for the examination of specimens removed from patients with gastric carcinoma: A basis for checklists. Members of the Cancer Committee, College of American Pathologists, and the Task Force for protocols on the examination of specimens from patients with gastric cancer. Arch Pathol Lab Med 122:9-14, 1998. 9448011

[8] Hammond ME, Compton CC: Protocols for the examination of tumors of diverse sites: Introduction. Cancer Committee of the College of American Pathologists. Arch Pathol Lab Med 124:13-16, 2000. 10629124 10.5858/2000-124-0013-PFTEOT

[9] Rosen RD, Sapra A: TNM Classification. Treasure Island, FL, StatPearls. 2021

[10] Cancer Protocol Templates: The College of American Pathologists. https://www.cap.org/protocols-and-guidelines/cancer-reporting-tools/cancer-protocol-templates

[11] https://cap.objects.frb.io/protocols/cp-femalereproductive-uterine-cervix-18protocol-4100.pdf

[12] https://documents.cap.org/protocols/cp-endocrine-thyroid-19-4100.pdf

[13] United Nations Sustainable Development Goals Knowledge Platform: Transforming our world: The 2030 agenda for sustainable development. https://sustainabledevelopment.un.org/post2015/transformingourworld

[14] Forman D, Ferlay J: The global and regional burden of cancer, in Stewart BW, Wild CP (eds): World Cancer Report 2014. Lyon, France, International Agency for Research on Cancer, pp 16-53

[15] Bray F, Znaor A, Cueva P: Planning and Developing Population-Based Cancer Registration in Low- and Middle-Income Settings. International Agency for Research on Cancer (IARC) Technical Publication No. 43, Lyon, France, WHO Press, 2014 (revised 2015), viii 33502836

[16] Srigley J, McGowan T, Maclean A, et al: Standardized synoptic pathology reporting: A population-based approach. J Surg Oncol 99:517-524, 2009 19466743 10.1002/jso.21282

[17] Lankshear S, Srigley J, McGowan T, et al: Standardized synoptic cancer pathology reports—so what and who cares? A population-based satisfaction survey of 970 pathologists, surgeons, and oncologists. Arch Pathol Lab Med 137:1599-1602, 2013 23432456 10.5858/arpa.2012-0656-OA

[18] Structured Pathology Reporting Of Cancer 2015-16. Final Report to Australian Government Department of Health. Royal College of Pathologists of Australasia, July 31, 2016. https://www1.health.gov.au/internet/main/publishing.nsf/Content/69FD4ADB686B4439CA25811B001DB252/$File/SPRC%20Final%20Report%202016%20Web%20.pdf

[19] Cancer Datasets and Tissue Pathways. The Royal College of Pathologists. 2021. https.//www.rcpath.org/profession/guidelines/cancer-datasets-and-tissue-pathways.html

[20] Published Datasets. International Collaboration on Cancer Reporting/Datasets. www.iccr-cancer.org/datasets

[21] Orah NO, Anunobi CC, Ojewola RW: Synoptic versus narrative reporting of prostate biopsies at a tertiary healthcare institution: Challenges, successes and expectations. Sultan Qaboos Univ Med J 17:319-323, 2017 10.18295/squmj.2017.17.03.010 PMC5642362 29062555

